# Supplementary material for: Pathological Features and Genomic Characterization of an Actinobacillus equuli subsp. equuli Bearing Unique Virulence-Associated Genes from an Adult Horse with Pleuropneumonia
Source: Pathogens. 2023 Jan 31;12(2):224. doi: 10.3390/pathogens12020224 (PMC9962156; doi:10.3390/pathogens12020224)
Supplement: Supplementary file 1 [file pathogens-12-00224-s001.zip › pathogens-2129028-supplementary.pdf]

Table S1. *Actinobacillus* species used in this study and their characteristics.

	**Invasive/ non-invasive species**	**Species**	**Strain**	**GenBank Accession**	**Host**	**Source**
1	Invasive species	*A. capsulatus*	DSM 19761	ARFN00000000.1	Rabbit	Joint
2		*A. equuli* subsp. *equuli*	ATCC 19392	NZ_CP007715.1	Horse	Unknown
3		*A. lignieresii*	NCTC4189	NZ_UFRM01000001	Cattle	Glands
4		*A. pleuropneumoniae*	NCTC10976	NZ_LR134515.1	Pig	Unknown
5		*A. seminis*	NCTC10851	NZ_UFSB01000001	Sheep	Semen
6		*A. suis*	ATCC33415	NZ_CP009159.1	Pig	Septicemia
7		*A. ureae*	NCTC10220	NZ_UFSD01000002.1	Human	Unknown
8	Non-invasive species	*A. delphinicola*	NCTC12871	NZ_LR134510.1	Porcine	Lung
9		*A. indolicus*	46K2C	NZ_CABFKH010000001.1	Pig	Upper respiratory tract
10		*A. minor*	NM305	ACQL00000000.1	Unknown	Unknown
11		*A. porcinus*	NM319	NZ_CABFKI010000029	Pig	Upper respiratory tract
12		*A. porcitonsillarum*	9953L55	NZ_CP029206.1	Pig	Unknown
13		*A. succinogenes*	GXAS137	NZ_NHRD00000000	Bovine	Rumen

Table S2. Shared and strain specific virulence-associated genes detected in *A. equuli* subsp. e*quuli* 4524 and ATCC 19392 strains

Virulence Factor	***A. equuli* subsp. *equuli* strain 4524 (JAPHVQ000000000)** Gene	Start	End	**Homologue gene detected in *A. equuli* subsp. *equuli* ATCC 19392 (NZ_CP007715)**	**Shared genes/ Strain specific genes**	**Nucleotide Identity (%)**	**Amino acid Identity (%)**	**Comments**
Iron acquisition	NA	NA	NA	*TonB-dependent siderophore receptor*	Strain specific genes	NA	NA	
	TonB-dependent receptor plug domain protein	15177	18164	NA		NA	NA	
	FhuD	127505	128386	NA		NA	NA	
	FhuB	128386	130335	NA		NA	NA	
	mrp	4188	5285	*apbC*	Shared genes	98.27	100	
	fepD	17374	18324	*fepD*		97.48	99.37	
	hutW	17463	19280	*hutW*		97.14	98.84	
	Hemin receptor	19211	21451	TonB-dependent receptor		98.53	99.46	
	fbpB	24003	26066	*fbpB*		98.64	99.56	
	cirA	45439	47415	*cirA*		98.99	99.09	
	fhuC	45811	46575	*ATP-binding cassette domain-containing protein*		99.48	100	
	fepB	46575	47528	*fepB*		98.64	99.06	
	fepD	47522	49468	*FhuB*		95.94	97.84	
	petE	49064	49495	*petE*		99.07	99.31	
	znuA	49397	50293	*znuA*		97.96	99.28	
	Outer membrane ferric hydroxamate receptor	49522	51609	*TonB-dependent siderophore receptor*		98.18	99.28	
	fepD	50288	51325	*fepD*		97.11	98.55	
	znuC	50295	51050	*znuC*		95.4	98.8	
	erpA	70275	70616	*erpA*		95.03	99.12	
	iscC	74520	75746	*iscS*		95.93	99.51	
	iscU	75879	76262	*iscU*		98.44	100	
	iscA	76403	76726	*iscA*		96.3	100	
	fdx	80441	80782	*fdx*		98.54	100	
	iscX	80782	80976	*iscX*		99.49	100	
	TonB-dependent receptor	81061	83778	*Lactoferrin/transferrin family TonB-dependent receptor*		99.68	99.78	

	Iron-regulated protein FrpC	100034	101014	*Hypothetical protein*		93.03	95.77	
	yddA	105860	107644	*yddA*		95.19	97.48	
	fbpB	110130	112130	*fbpB*		94	96.4	
	afuA	112262	113293	*ABC transporter substrate-binding protein*		95.16	97.97	
	ceuD	145185	145943	*ceuD*		97.1	98.81	
	ceuC	145946	146893	*ceuC*		97.57	98.1	
	cirA	146541	148835	*cirA*		98.95	98.95	
	ceuB	146886	147782	*ceuB*		99.44	99.67	
	cysG	188818	190278	*cysG*		95.62	97.54	
	efeB	192983	194173	*efeB*		99.24	98.74	
	fur	216404	216853	*fur*		99.56	100	
	cirA	261290	263803	*cirA*		96.86	98.69	
	cyaA	284521	284826	*cyaY*		99.67	100	
	cirA	298547	301384	*cirA*		99.26	99.68	
	cirA	305007	307307	*cirA*		99.46	99.59	
	znuB	395938	396768	*znuB*		98.19	99.28	
	exbB	474019	474471	*exbB*		94.92	100	
	exbD	474516	474905	*exbD*		99.23	100	
	Protein TonB	474915	475760	*Energy transducer TonB*		97.4	98.94	
LPS	NA	NA	NA	Wzz/FepE/Etk N-terminal domain-containing protein	Strain specific genes	NA	NA	
	NA	NA	NA	*rfaJ*		NA	NA	
	NA	NA	NA	*Glycosyltransferase family 4 protein*		NA	NA	
	glycosyltransferase family 2 protein	23864	25705	NA		NA	NA	
	rfaF	220335	221534	NA		NA	NA	
	fabZ	154332	154796	*fabZ*	Shared genes	93.98	99.35	
	Udp-glcnac--lipooligosaccharide n-acetylglucosaminyl glycosyltransferase	463019	463717	*Glycosyltransferase family 25 protein*		94.42	100	
	glf	61311	62462	*glf*		55.01	44.63	The nucleotide and amino acid sequences of *glf* gene of strain 4524 show 89.93% and 94% similarity, respectively, to that of

						homologue gene in *A. pleuropneumoniae* strain 3906
glmU	65991	67355	*glmU*	96.56	98.9	
kdsB1	468431	469183	*kdsB*	96.55	99.2	
lptD	55830	58160	*lptD*	90.87	96.39	
lptE	107788	108282	*lptE*	95.76	98.78	
lptC	174746	175369	*lptC*	99.68	99.51	
lptA	175366	175866	*lptA*	95.32	97	
lptF	265316	266419	*lptF*	93.3	99.18	
lptG	266420	267502	*lptG*	95.94	99.72	
murG	54675	55730	*murG*	98.2	99.14	
kdsC	16446	16982	*kdsC*	97.21	98.88	
wbaP	58126	59544	*wbaP*	65.04	63.58	The nucleotide and amino acid sequences of *wbaP* gene of strain 4524 show 96.83% and 94% similarity, respectively, to that of homologue gene of *A. suis* strains NCTC12996 and ATCC 33415
Pilin glycosyltransferase	118954	119595	*Glycosyltransferase family 4 protein*	89.04	81.94	
Glycosyltransferase	146773	147561	*Glycosyltransferase family 2 protein*	96.58	99.24	
Beta-galactoside alpha-2,3-sialyltransferase	328450	329379	*Glycosyltransferase family 52*	99.03	99.35	
Lipooligosaccharide N-acetylglucosamine glycosyltransferase	329383	330153	*Glycosyltransferase family 25 protein*	94.03	94.94	
wcaA	331344	332225	*wcaA*	98.53	97.95	
wcaA	165271	166029	*wcaA*	97.63	99.2	
rfaF	166998	168035	*rfaF*	96.24	99.71	

	Glycosyl transferase family protein	179116	179904	*Glycosyltransferase family 8 protein*		85.62	88.21	
	wcaA	35490	36281	*Glycosyltransferase family 8 protein*		86.82	89.26	
	rfaF	220335	221534	*rfaF*		95.25	97.75	
	rfaF	306770	307816	*rfaF*		99.62	99.71	
	Glyco-trans-2-like domain-containing protein	385209	386027	*Glycosyltransferase family 2 protein*		89.01	91.94	
	Udp-glcnac--lipooligosaccharide n-acetylglucosaminyl glycosyltransferase	463019	463717	*Udp-glcnac--lipooligosaccharide n-acetylglucosaminyl glycosyltransferase*		94.42	95.27	
	rfaJ	299322	300164	*rfaJ*		89.21	90.03	
	wcaA	35490	36281	*wcaA*		89.55	92.04	
	rfaJ	345	1190	*rfaJ*		99.88	100	
	glycosyltransferase family 2 protein	23864	25705	*Glycosyltransferase family 2 protein*		37.49	20.22	The amino acid sequence of glycosyltransferase family 2 protein was 99.35% identical to that of holomgue protein in *A. ureae*
	msrQ	5298	5894	*msrQ*		99.33	98.995	
	gmhA	35386	35970	*gmhA/ipcA*		98.8	99.48	
	kdtA	298707	299987	*waaA*		91.65	95.08	
	yciS	128557	128847	*yciS*		93.47	98.96	
	lapB	128847	130034	*lapB*		97.81	98.99	
	waaF	78287	79324	*waaF*		94.99	96.53	
	waaC	79385	80359	*rfaC*		95.94	96.25	
	rfbX	327242	328450	*rfbX*		97.93	99.25	
	rfbB	56970	58043	*rfbB*		92.53	95.25	
	glf	61311	62462	*glf*		55	43.43	The nucleotide and amino acid sequences of *glf* gene of strain 4524 showed 89.93% and 94% similarity, respectively, to that of homologue gene in *A. pleuropneumoniae* strain 3906
CPS	NA	NA	NA	*Glycosyl transferase*		NA	NA	

	Putative glycosyltransferase YkoT	65531	66496	NA	Strain specific genes	NA	NA
	murB	67774	68802	*murB*	Shared genes	99.03	98.83
	murC	53196	54623	*murC*		94.19	98.74
	murD	57061	58371	*murD*		94.51	98.17
	murE	61169	62674	*murE*		97.34	97.61
	murF	59650	61044	*murF*		96.77	98.71
	wecC	9986	11254	*wecC*		96.69	98.1
	riml	9363	9986	*rffC*		95.99	96.63
	cysA	70739	71554	*elaA*		94.42	95.83
	wecE	8214	9347	*rffA*		98.41	98.94
	rfbA	56101	56973	*rfbA*		97.25	97.6
	gutQ	117062	117997	*gutQ*		88.68	94.23
	Glycosyl transferase	120713	121546	*Glycosyl transferase*		87.65	88.49
	galE	124052	125068	*galE*		90.86	98.52
	murA	141521	142798	*murA*		96.48	98.6
	rpiR	155869	156741	*rpiR*		97.59	100
	nagC	19616	20488	*nagC*		97.02	98.28
	rfaF	219155	220342	*rfaF*		94.11	96.71
	wza	116057	117214	*wza*		93.26	98.44
Miscellaneous	NA	NA	NA	*ShlB/FhaC/HecB family hemolysin secretion/activation protein*	Strain specific genes	NA	NA
	NA	NA	NA	*ShlB/FhaC/HecB family hemolysin secretion/activation protein*		NA	NA
	pgaC	172303	173538	NA		NA	NA
	dps	96363	96932	*dps*	Shared genes	97.72	99.47
	UreD	54034	54846	*UreD*		97.66	100
	UreG	55032	55667	*UreG*		94.81	99.53
	UreF	55688	56398	*UreF*		98.39	100
	UreE	56383	56946	*UreE*		99.47	100
	UreA	59925	60227	*UreA*		99.67	100
	UreB	59493	59798	*UreB*		100	100
	UreC	57763	59481	UreC		98.37	99.48
	Recombination protein 2	94843	97137	*DNA internalization-related competence protein ComEC/Rec2*		95.45	94.64

	fhaC	441089	442843	fhaC		91.69	95.72	
	gstA	219432	220058	*gstA*		92.66	99.04	
	hypothetical protein	57019	57306	*gstA*		97.92	98.96	
	gstA	19439	20056	*gstA*		98.38	99.51	
	pgaB	173552	175324	*pgaB*		98.93	99.32	
	pgaA	175338	177758	*pgaA*		94.63	96.9	
OMP	*ompC*	93317	94447	*ompC*	Shared genes	95.5	96.81	
	Outer membrane protein P2 (OMP P2)	169146	170249	*Outer membrane protein P2 (OMP P2)*		99.18	99.45	
	47 kDa outer membrane protein	72215	73555	*Outer membrane protein transport protein (OMPP1/FadL/TodX)*		94.77	93.88	
	ompW	250909	251562	*ompW*		99.24	100	
Salicylic acid	*nanE*	20498	21187	*nanE*	Shared genes	97.54	97.82	
	nanA	18717	19595	*nanA*		98.18	99.31	
RTX toxin	*apxIB*	31379	33502	sunT	Shared genes	64.78	64.55	The nucleotide and amino acid sequences of *apxIB* gene of strain 4524 showed 99.72% and 99.86% similarity, respectively, to aqxB gene of *A. equuli subsp. haemolyticus* strain CCUG 19799
Auto-transporter	*Autotransporter adhesin*	74395	78480	YadA-like family protein	Shared genes	87.9	55.8	The nucleotide and amino acid sequences of *Autotransporter adhision* gene of strain 4524 showed 95.25% and 96% similarity, respectively, to that of homologue gene in *A. suis* strain ATCC 33415
